# An approach for an enhanced anticancer activity of ferulic acid-loaded polymeric micelles via MicroRNA-221 mediated activation of TP53INP1 in caco-2 cell line

**DOI:** 10.1038/s41598-024-52143-y

**Published:** 2024-01-24

**Authors:** Nabila M. Sweed, Marwa H. S. Dawoud, Nora M. Aborehab, Shahira M. Ezzat

**Affiliations:** 1 Department of Pharmaceutics, Faculty of Pharmacy, October University for Modern Sciences and Arts, Giza, Egypt; 2 Department of Biochemistry, Faculty of Pharmacy, October University for Modern Sciences and Arts (MSA), Giza, 12451 Egypt; 3 Pharmacognosy Department, Faculty of Pharmacy, Cairo University, Kasr El-Aini Street, Cairo, 11562 Egypt; 4 Department of Pharmacognosy, Faculty of Pharmacy, October University for Modern Sciences and Arts (MSA), Giza, 12451 Egypt

**Keywords:** Cancer, Drug discovery, Plant sciences

## Abstract

Ferulic acid (FA) has powerful antioxidant and antitumor activities, but it has low bioavailability owing to its poor water solubility. Our aim is to formulate polymeric mixed micelles loaded with FA to overcome its poor solubility and investigate its potential anticancer activity via miRNA-221/TP53INP1 axis-mediated autophagy in colon cancer. A D-optimal design with three factors was used for the optimization of polymeric mixed micelles by studying the effects of each of total Pluronics mixture (mg), Pluronic P123 percentage (%w/w), and drug amount (mg) on both entrapment efficiency (EE%) and particle size. The anticancer activity of FA and Tocopheryl polyethylene glycol 1000 succinate (TPGS) mixed micelles formula (O2) was assessed by MTT and flow cytometry. O2 showed an EE% of 99.89%, a particle size of 13.86 nm, and a zeta potential of − 6.02 mv. In-vitro drug release studies showed a notable increase in the release rate of FA from O2, as compared to the free FA. The (IC_50_) values for FA from O2 and free FA were calculated against different cell lines showing a prominent IC_50_ against Caco-2 (17.1 µg/ml, 191 µg/ml respectively). Flow cytometry showed that FA caused cell cycle arrest at the G2/M phase in Caco-2. RT-PCR showed that O2 significantly increased the mRNA expression level of Bax and CASP-3 (4.72 ± 0.17, 3.67 ± 0.14), respectively when compared to free FA (2.59 ± 0.13, 2.14 ± 0.15), while miRNA 221 levels were decreased by the treatment with O2 (0.58 ± 0.02) when compared to free FA treatment (0.79 ± 0.03). The gene expression of TP53INP1 was increased by the treatment with O2 compared to FA at P < 0.0001. FA-loaded TPGS mixed micelles showed promising results for enhancing the anticancer effect of FA against colorectal cancer, probably due to its enhanced solubility. Thus, FA-loaded TPGS mixed micelles could be a potential therapeutic agent for colorectal cancer by targeting miRNA-221/TP53INP1 axis-mediated autophagy.

## Introduction

Colorectal cancer (CRC) is the third most common cancer and the fourth leading cause of cancer‑associated death worldwide^1^. Resection and chemotherapy are the primary therapeutic methods used for colon cancer control. Chemotherapy is also the conventional adjuvant therapy following surgery for advanced colon cancer^2^.

Ferulic acid (4-hydroxy-3-methoxycinnamic acid), a phenolic compound found in many fruits and vegetables, is found to be a powerful antioxidant, anti-tumor, and anti-inflammatory agent. The antioxidant effect is due to its ability to neutralize superoxide, nitric oxide, and hydroxyl radicals, which are known to cause oxidative damage to cell membranes and DNA^3–5^.

Previous studies have reported that FA caused inhibition to the expression and activity of cytotoxic enzymes, such as inducible nitric oxide synthase, caspases and cyclooxygenase-2. Therefore, it can be used in the treatment of many cancer types such as stomach, breast, colon, liver, prostate, lung, osteosarcoma, and tongue^3–6^.

Ferulic acid has low oral bioavailability (9–20%) owing to its poor solubility, as well as its rapid metabolism by the liver. Several approaches have been used to overcome these problems, such as solid lipid nanoparticles^7^, chitosan nanoparticles^8^ and poly (lactic-co-glycolic acid) nanoparticles^9^. Incorporation of FA into polymeric micelles could be another good strategy for the enhancement of FA solubility and efficacy^9^.

Polymeric micelles (PM) are made up of amphiphilic polymers that self-assemble into nanostructures. The inner core of PMs is hydrophobic, which is used to solubilize drugs with poor water solubility, and the outer shell is hydrophilic, which isolates the encapsulated drug from the external medium, to achieve better drug stability^10,11^.

The size of the polymeric micelles ranges from 10 to 100 nm, and thus they are large enough to escape the elimination via glomerular filtration, but small enough to escape scavenging by the reticuloendothelial system. This in turn, allows the micelles to circulate for a longer time^12^.

It has been reported that polymeric micelles increase the drug cellular uptake, and provide an alternative pathway of internalization (endosomes). This in turn, makes the use of PM of great importance, especially when the treatment is greatly affected by drug efflux mechanisms related to multi-drug resistance^13^.

Pluronics are amphiphilic triblock copolymers, which are composed of hydrophilic polyethylene oxide (PEO) and hydrophobic polypropylene oxide (PPO) blocks (PEO–PPO–PEO)^14,15^. They are widely used in the preparation of PM due to their potential solubilizing effect, and their inhibition of P-glycoprotein (P-gp) efflux, in addition to their increased circulation time^16^.

D-Tocopheryl polyethylene glycol 1000 succinate (TPGS) is a water-soluble derivative of Vitamin E and polyethylene glycol 1000, which is approved by the FDA as a safe pharmaceutical additive. It has a high solubilizing capacity, and is considered to be a good absorption enhancer. TPGS is commonly used in formulating drugs used for cancer therapy. This is because TPGS has been reported to act as a selective potent proapoptotic agent for cancer cells. In addition, TPGS enhances the cellular uptake of anticancer drugs, prolongs their blood circulation time, enhances their transport across the cellular barrier, and inhibits P-gp efflux pump^17^.

Small non-coding RNAs called microRNAs (miRNAs) have 20–22 nucleotides and are involved in several biological activities. Numerous malignancies, including brain, lung, breast, liver, prostate, and colorectal cancer, have been linked to the development and advancement of miRNAs. Several characteristics of cancer cells are influenced by miRNAs, which act as tumor suppressors or oncogenes by targeting the 30UTR of target genes. These characteristics include mediating cell proliferation and migration, autophagy, apoptosis, metabolic shift, epithelial-mesenchymal transition, and radiosensitivity^18^.

Autophagy, a cellular self-degradative mechanism that mobilizes intracellular nutritional resources, is crucial for cell survival under stressful circumstances. However, hyperactive autophagy can result in programmed cell death that is non-apoptotic. Numerous human disorders, such as cancer, are linked to the dysregulation of this mechanism. Autophagy plays a multifaceted role in cancer and exhibits either oncogenic or tumor-suppressor activity that can either promote tumor growth or repress it^19^.

Previous research indicates that autophagy in cancer is greatly influenced by post-transcriptional and translational control mediated by microRNAs. Other research suggests that miR221 inhibits autophagy activity in heart failure by modifying the p27/CDK2/mTOR axis. Autophagy mediated by hypoxia/reoxygenation is reported to be inhibited by upregulating miR-221^1^.

This study aims to incorporate ferulic acid into polymeric mixed micelles made of Pluronics and TPGS, in an attempt to enhance its solubility and stability, and to investigate its potential anti- cancer activity via miRNA-221/TP53INP1 axis-mediated autophagy on Caco-2 cancer cell line.

## Materials and methods

### Chemicals and reagents

Pluronic F127 (F127), Pluronic P123 (P123), and D-α-tocopheryl polyethylene glycol 1000 succinate (TPGS) were purchased from Sigma-Aldrich Chemical Co. (St. Louis, IL, USA). All other chemicals were of analytical grade and were used as received. Diaion HP-20 AG for column chromatography (75–150 µm, Mitsubishi Chemical Industries Co. Ltd) and Sephadex LH-20 (Pharmacia, Stockholm, Sweden) were used for column chromatography (CC). Thin-layer chromatography (TLC) was performed on silica gel GF_254_ precoated plates (Fluka), using methylene chloride/methanol (80:20 v/v) as a solvent system. Bruker NMR was used for ^1^H NMR (400 MHz) and ^13^C NMR (125 MHz) measurements. The NMR spectra were recorded in DMSO and chemical shifts are given in δ (ppm) relative to TMS as an internal standard.

### Isolation of ferulic acid

The n-butanol fraction was prepared from *Liatris spicata* (L.) (10 g) as mentioned in our published data^20^ and it was portioned on a diaion HP-20 AG (250 g, 5 × 120 cm) using water, followed by water/methanol (1:1 v/v) and finally with 100% methanol. The water/methanol fraction was then purified several times over Sephadex LH—20 columns using water/methanol (1:1 v/v) as eluent to give white crystals (120 mg, R_f_ = 0.72 on TLC in methylene chloride/methanol (80:20 v/v)).

For identification of the compound, it was analyzed using ^1^HNMR and ^13^CNMR analysis, where the following data were obtained:

^1^H NMR (400 MHz, DMSO): *δ* = 3.82 (s, 3H, –OCH_3_), 6.35 (d, *J* = 15.8 Hz, 1H, 8-H), 6.79 (d, *J* = 8.1 Hz, 1H, 5-H), 7.07 (dd, *J* = 8.1, 1.5 Hz, 1H, 6-H), 7.28 (d, *J* = 1.5 Hz, 1H, 2-H), 7.48 (d, *J* = 15.8 Hz, 1H, 7-H).^13^C NMR (125 MHz, DMSO): *δ* = 56.13 (–OCH3), 126.2 (C-1), 111.6 (C-2), 148.3 (C-3), 149.5 (C-4), 116.0 (C-5), 123.2 (C-6), 144.9 (C-7), 115.9 (C-8), 168.4 (–COOH).

### Experimental design

A D-optimal design with three factors was used to study the effects of the different factors on the polymeric mixed micelles characteristics, using Design Expert® software (Version 10, Stat-Ease Inc., Minneapolis, MN). The independent variables were X_1_: total Pluronics mixture (mg), (X_2_): Pluronic P123 percentage (%w/w), and (X_3_): drug amount (mg), whereas the dependant variables were the entrapment efficiency percentage (EE%) (Y_1_) and the particle size (Y_2_), as shown in Table 1.

**Table 1:** D-optimal design of FA polymeric mixed micelles, and the composition of the 20 formulae.

Independent variables	Low level (−1)	High level (1)			
X_1_: total pluronics (mg)	200	400			
X_2_: pluronic P123 (%)	10	90			
X_3_: drug amount (mg)	5	10			

Dependent variables	Constraints				
Y_1_: entrapment efficiency (%)	Maximize				
Y_2_: particle size (nm)	Minimize				

Formula code	X_1_: total pluronics (mg)	X_2_: pluoronic P 123 (%)	X_3_: drug amount (mg)	Y_1_ = EE (%)	Y_2_ = particle size (nm)
F1	200	90	10	81.60 ± 0.47	20.50 ± 0.12
F2	400	55.48895	6.825	77.67 ± 0.34	24.07 ± 0.11
F3	400	62	10	66.33 ± 0.23	24.48 ± 0.22
F4	200	90	10	84.15 ± 0.45	20.00 ± 0.25
F5	200	10	5	100.00 ± 0.33	27.70 ± 0.29
F6	200	42	8	84.55 ± 0.26	25.12 ± 0.25
F7	200	90	5	100.00 ± 0.22	20.44 ± 0.30
F8	280	10	7	97.00 ± 0.21	27.07 ± 0.36
F9	300	82	5.5	82.56 ± 0.34	20.85 ± 0.38
F10	319	90	7.975	95.29 ± 0.17	21.50 ± 0.19
F11	400	10	6.775	98.01 ± 0.27	28.29 ± 0.29
F12	400	10	10	80.92 ± 0.37	27.96 ± 0.26
F13	310	46	8.5	73.16 ± 0.43	25.83 ± 0.44
F14	400	90	5	82.00 ± 0.21	19.93 ± 0.33
F15	400	90	5	82.00 ± 0.22	20.01 ± 0.12
F16	320	42.4	5	67.68 ± 0.23	27.41 ± 0.25
F17	320	42.4	5	67.20 ± 0.33	27.10 ± 0.22
F18	200	90	5	99.20 ± 0.32	20.10 ± 0.12
F19	200	10	5	97.00 ± 0.38	26.70 ± 0.11
F20	272	10	10	72.00 ± 0.28	26.36 ± 0.34

Data are given as mean ± SD (n = 3).

According to the chosen D-optimal design, 20 formulae were prepared and their composition is shown in Table 1. Analysis of variance (ANOVA) was carried out to estimate the significance of the model and terms, where probability at p-values level (p < 0.05), were denoted significant^21^.

#### Preparation of ferulic acid mixed micelles

Thin film hydration method was used to prepare ferulic acid mixed micelles. First, the required amount of FA was accurately weighed, according to Table 1, and dissolved in 10 ml methanol. The drug solution was then placed into a round-bottom flask (250 ml) containing the Pluronics mixture; composed of Pluronic F127 and Pluronic P123, and sonicated until the Pluronics mixture was completely dissolved. The solvent was then subjected to evaporation under vacuum using a rotary vacuum evaporator (Heidolph, Germany) till a dry thin film was formed. Ten ml of distilled water was used for the hydration of the film, and rotation was continued for 1 h under normal pressure (1 atmosphere). Filtration was then done using a Millipore® filter of 0.45µm, to remove the free unentrapped drug, and the ferulic acid polymeric mixed micelles was then stored at 4 ºC^12^.

#### Optimization of the FA micelles

An optimized formula of FA mixed micelles was suggested by the Design Expert® software, by applying the following constraints: maximizing the EE%, and minimizing the PS, as shown in Table 1. The optimized formula (O1) was then prepared and characterized to check the validity of the experimental design.

#### Preparation of TPGS mixed micelles

The optimized micelles formula was selected to be further modified using TPGS, where the ratio of the total Pluronics to TPGS was 1:1. The formula (O2) was prepared using the same procedure for the preparation of the Pluronic micelles, where the TPGS was dissolved with the Pluronics mixture^22^.

#### Characterization of the prepared micelles

##### Determination of the entrapment efficiency (EE) %

A known volume of the micellar dispersion was dissolved in methanol and measured spectrophotometrically at the pre-determined λ_max_ 314 nm (UV-1700, Shimadzu, Tokyo, Japan). The concentration of FA was determined using the pre-constructed calibration curve in methanol^12^. Each experiment was conducted in triplicate. The EE % was calculated using Eq. (1):

1 $${\text{EE }}\% = {\text{ weight of the drug in micelles}}/{\text{weight of the drug used in the preparation of micelles }} \times {1}00$$

##### Determination of the micelles’ particle size and polydispersity index (PDI)

The micelles’ particle size and the polydispersity index (PDI) were measured for all the prepared formulae by dynamic light scattering technique, using zetasizer (Malven Zetasizer version 6.20 serial number: MAL 104 4595, Worcestershire, United Kingdom), at a fixed angle of 173°. The samples were measured without any dilution. All measurements were done in triplicate^23^.

##### Determination of zeta potential

The zeta potential for the optimized FA micelles was measured using a zeta sizer, by determining the particle electrophoretic velocity using zetasizer. The samples were measured without any dilution. All measurements were done in triplicate^22,23^.

### Differential scanning calorimetry (DSC) measurement

Using differential scanning calorimetry (DSC-60 Shimadzu, Tokyo, Japan), thermograms of ferulic acid, TPGS mixed micelles, and blank TPGS mixed micelles were recorded. A sample of each of ferulic acid, TPGS mixed micelles (O2), and blank TPGS mixed micelles was put in an aluminium pan with a heating rate of 10 °C/min from 50 to 400 °C and the sample cell was constantly purged with nitrogen at a flow rate of 40 mL/min^24,25^.

### Fourier transform infrared spectroscopy (FTIR) measurement

Fourier transform infrared spectra of FA, TPGS mixed micelles (O2), and blank TPGS mixed micelles were recorded on a FTIR spectrophotometer (JASCO FTIR-8400, Japan) after mixing with dry potassium bromide. Each sample was scanned in the range of 4000–400 cm^−1^ at a resolution of 2 cm^−1^^26^.

### Transmission electron microscopy (TEM)

For the examination of the morphology of the prepared micelles, a drop of the micellar dispersion was placed on a copper grid, negatively stained with 2% phosphotungstic acid, and left to dry. After that, the film was examined using TEM (Ted Pella, Redding, CA), and different photos were captured^12,16^.

### Determination of critical micelle concentration (CMC)

The iodine UV spectroscopy method was used to determine the CMC of the optimized TPGS-Pluronics mixed micelles formula, as well as the CMC of Pluronic F127, Pluronic P123, and TPGS micelles in distilled water^27^. In order to prepare a standard potassium iodide/iodine solution, one gram of potassium iodide in addition to 0.5 g of iodine were dissolved in 50 ml distilled. Then, samples of each of Pluronic F127, Pluronic P123, TPGS, and the TPGS mixed micelles formula were prepared in concentrations ranging from 0.00001 to 0.1%. After that, 100 µl of potassium iodide/iodine solution was added to each prepared sample. Iodine was used as a hydrophobic probe because solubilized iodine (I2) tends to reside in the hydrophobic regions of the P123/F127 copolymer. I3 is converted to I2 from excess KI in solution to maintain a saturated concentration of I2 in water.

The prepared samples were incubated in a dark place for 12 h at room temperature, after which the UV absorbance of each concentration was measured at 366 nm. Experiments were done in triplicate. In order to determine the CMC, the absorbance was plotted against log (concentration) of the prepared samples. The concentration where a sharp increase in absorbance was observed, corresponds to the CMC^22^. The lower the CMC value, the higher the stability of the micelles upon dilution.

### Solubility of FA in water and polymeric micellar solution

Ferulic acid powder was added in excess amount to 2 ml of distilled water and placed in a thermostatically controlled shaker (incubator shaker, ZHWY-2102C, Shanghai, China) at 100 rpm at 25 °C for 72 h, followed by centrifugation at 15,000*g* for 15 min. Filtration of the supernatant was done using a 0.2 μm Millipore ®filter, and the amount of FA in the filtrate was measured spectrophotometrically at λ_max_ 309 nm. In order to determine the solubility of each of optimized mixed micelles (O1) and TPGS mixed micelles formula (O2), 20 μl of the each micellar solution was suitably diluted with methanol, and the amount of FA was determined spectrophotometrically at 314 nm, and the solubility of FA in the micellar solution was calculated^28^.

### In-vitro drug release

The in-vitro drug release was done using the dialysis bag method. Briefly, 4 ml of each of the optimized mixed micelles (O1) and the TPGS mixed micelles (O2) (each containing 3 mg FA), and standard FA suspension (prepared by suspending 3 mg FA in 4 ml of distilled water), were placed in dialysis bags (Spectra/Por® Molecular Porous Membrane Tubing, flat width 23 mm, diameter 14.6 mm, MWCO 6–8000; SpectruLaboratories Inc., Rancho Dominguez, CA, USA). The bags were inserted into containers filled with 100 ml of the release medium (phosphate buffer saline of pH 7.4 containing 0.5% Tween 80)^28^**,** and placed in a thermostatically controlled shaking water bath at 37 ± 0.5 °C and 100 rpm. Aliquots of 2 ml were withdrawn at 5, 10, 15, 30, 60, 120, 180, 240, 300, and 360 min, and replaced with equal volumes of fresh release medium. Filtration of the samples was done using a Millipore® membrane filter (0.45 µm), and measured spectrophotometrically at λ_max_ = 309 nm. The concentration of FA was determined using the pre-constructed calibration curve in phosphate buffer saline of pH 7.4 containing 0.5% Tween 80. The percentage cumulative release of FA at different time intervals was calculated using Eq. (2):

2 $$\% {\text{ Cumulative release }} = \, \left( {{\text{Amount of FA in the medium}}/{\text{Amount of FA in the micelles}}} \right) \, \times { 1}00$$

### Stability studies

In order to test the storage stability of the TPGS mixed micelles (O2), the micelles were incubated at 4 °C and relative humidity of 55–60% for three months^29^ and then both the micelles’ particle size and the entrapment efficiency % were measured after 3 months^30^.

### Biological screening

#### Cell culture and reagents

Vero cells (normal kidney cells), human colon cancer cell line (Caco-2), human pancreatic cancer (PANC-1), human breast adenocarcinoma (MCF-7), human hepatocellular carcinoma (HepG2), and human lung adenocarcinoma (A549) were obtained from (Holding Company for Biological Products and Vaccines VACSERA; Giza, Egypt), all cell lines were cultured in RPMI 1640 medium (Lonza, Switzerland), with 10% fetal bovine serum (Gibco), 1% penicillin, and 1% streptomycin (Sigma Aldrich, USA) supplied in all media. In a humidified cell incubator with a 5% CO_2_ atmosphere, cells were kept at 37 °C.

#### MTT assay

The MTT is a colorimetric assay that is used to determine the IC50 of ferulic acid (FA), TPGS mixed micelles formula (O2), and blank TPGS mixed micelles (B). To generate a full monolayer sheet, all cell lines were inoculated on 96 well tissue culture plates at the density of (1 × 10^5^ cells/ml (100 µl/well)), and incubated at 37 °C for 24 h. After the formation of a merged sheet of cells, the 96 well microtiter was decanted from the growth medium and replaced with different concentrations of compounds (FA, Q2 and B) (μg/ml) for 48 h, and the plate was incubated at 37 °C. MTT solution (5 mg/ml in PBS) (BIO BASIC CANADA INC) was prepared, and added to each well for incubation for 4 h. NADPH-dependent cellular oxidoreductase enzyme reduces yellow 3-(4,5-dimethylthiazol-2-yl)-2,5diphenyl-tetrazolium bromide (MTT) to formazan product (purple colour) in the viable cells. For the solubilization of the formazan crystals; 200 μl DMSO was added into each well after the removal of the media and a microplate reader (mindray, MR-96A) was used to measure the optical density of the formazan product at 560 nm. The average of the three independent experiments was used to calculate the results.

The percentage of viability was calculated by dividing the optical density (OD) of the sample (cell line with the different concentrations of the drugs) and the optical density (OD) of the control (cell line without the drugs) by using Eq. (3):

3 $$\% {\text{ of cell viability}} = {\text{ OD of Sample}}/{\text{OD of Control }} \times { 1}00$$

### Cell cycle analysis and apoptosis assay by flow cytometry

The well walls of cultured Caco-2 cells were left attached to them overnight. After incubation for 24 h, the cells were treated with IC_50_ of FA and O2 for 48 h, and rinsed 3 times with ice-cold Phosphate buffer saline. Cells were harvested from both samples. After that 500 μl of binding buffer was used for the reconstitution of the cells. In the dark for 5 min, 5 μl of both Annexin V- fluorescein isothiocyanate (FITC) and propidium iodide (PI) were added. (BD FACSCalibur, India; Becton Dickinson, Biosciences) Flow cytometry was used to detect Cell cycle distribution.

To identify the cells in different phases, a double staining kit (BioVision Research Products, USA) was used to stain the cells at room temperature with FITC and PI for 15 min in the dark.

### RNA extraction

Caco-2 cells were treated with IC_50_ of FA and O2 and allowed for incubation for 48 h. The RNeasy mini kit (Qiagen, GmbH, Germany) was used for the preparation of RNA samples according to the manufacturer’s instructions.

### Quantitative polymerase chain reaction (qPCR)

RT-qPCR was carried out using iScript ™ One-Step RT-PCR Kit with SYBR® Green (Bio-Rad Laboratories, CA) on Rotor-Gene Q real-time PCR cycler. The primers (Life Technologies) used for the target genes: Bax gene, CASP-3 gene, TP53INP1 gene and the housekeeping gene β-actin are listed in S1. Data from real-time PCR were used to calculate the relative expression of tested genes mRNA. For the normalization of all values; the β-actin gene was utilized. Finally, values were reported as fold change using Eq. (4):^31^

4 $$2^{{\Delta \Delta {\text{CT}}}} .$$

For miRNA normalization, the internal control (U6 RNA) was used and the primer set for the internal control was purchased from RiboBio Co., Ltd. All samples were run in triplicate and the relative expression of miR‑221 was quantified by the same method.

### Statistical analysis

All results were expressed as mean ± SD. A one-way ANOVA test was used to evaluate the statistical significance, wherever applicable, using Design-Expert 10.0.1.0 software (Stat-Ease Inc., USA), where statistical significance was considered at p ˂ 0.05 level.

Parameters of biological measurements were evaluated using the Graph Pad Prism® version 6 (California Corporation, USA). Values were expressed as the mean ± SD of the triplicates of each experiment. For normally distributed quantitative variables, a one-way ANOVA with multiple comparisons post hoc test was utilized. A statistically significant *P* value of less than 0.05 was acceptable.

### Statement of human and animal rights

This article does not contain any studies with human or animal subjects performed by any of the authors.

## Results

### Identification of ferulic acid

The ^1^H-NMR spectrum of FA showed the characteristic signals for 4-Hydroxy-3-methoxycinnamic acid (Ferulic acid) (Bunzel et al., 2005). This was characterized by two trans-olefinic protons at − 6.35 and 7.48 ppm with large coupling constant 15.8 Hz, assigned to H-8 and H-7, respectively. An ABX system was affirmed by these signals at − 6.79, 7.07 and 7.28 ppm, corresponding to H-5, H-6 and H-2, respectively. The methoxy group at C-3 was manifested by the singlet signal at − 3.82 ppm and integrated as three protons.

The ^13^C-NMR supported the nine characteristic carbon signals for 4-Hydroxy-3-methoxycinnamic acid with two oxy carbons at − 148.3 and 149.5 ppm (C-3 and C- 4), one carbonyl carbon at 168.4 ppm (C-9), two trans olefinic carbons at − 144.9 and − 115.9 ppm and finally, C-1, C-2, C-5 and C-6 were confirmed by signals at − 126.2, 111.6, 116.0 and 123.2 ppm respectively.

### Statistical analysis using D-optimal design

A D-optimal design was used to study the effects of both the total Pluronics mixture (X_1_), Pluronic P123% (X_2_), and drug amount (X3), on the EE% (Y_1_) and particle size (Y_2_), as listed in Table 1. The results for the EE % and the particle size are given in Table 1. The optimized formula was chosen to have a maximum EE % and a minimum particle size.

The chosen design supported a quadratic model for the measured responses, namely the EE %, and the particle size. The fitted models had a high correlation coefficient R^2^, an adjusted R^2^ in a reasonable agreement with the predicted R^2^, and adequate precision values (0.9950, 0.9905, 0.9801, 41.809, for EE %, and 0.9806, 0.9631, 0.9145, 18.944 for particle size, respectively).

ANOVA test was used to identify the significant terms. Model terms with p < 0.05 were considered statistically significant. In order to obtain a higher prediction R^2^, the non-significant model terms were removed. Reduced regression models for EE% and particle size were created by removing all insignificant variables except those necessary to keep hierarchy using the backward approach^32^. The regression equations obtained for the responses were as represented in Eqs. (5) and (6):

5 $$\begin{gathered} {\text{EE}}\% \, = \, 77.58 \, - 3.18 \, * \, X_{1} + \, 0.87 \, * \, X_{2} - 5.30 \, * \, X_{3} - 1.15 \, * \, X_{1} X_{2} + \, 4.60 \, * \, X_{1} X_{3} \hfill \\ + \, 1.62 \, * \, X_{2} X_{3} + \, 5.42 \, * \, X_{1}^{2} + \, 18.29 \, * \, X_{2}^{2} - 15.23 \, * \, X_{3}^{2} \hfill \\ \end{gathered}$$

6 $${\text{PS}} = 25.44 \, + \, 0.59 \, * \, X_{1} - 3.43 \, * \, X_{2} {-} \, 0.28 \, X_{3} - 0.538031 \, * \, X_{1} X_{2} + \, 0.621575 \, * \, X_{2} X_{3} - 1.3943 \, * \, X_{2}^{2}$$

A positive coefficient sign denotes a synergistic impact, while a negative coefficient sign denotes an antagonistic effect. The larger the coefficient, the more the influence of that factor on the studied response^33^.

The EE% for all the prepared formulae ranged from 66.33 to 100% as shown in Table 1, which show a high EE%.

As shown in Table 2, the total Pluronics mixture (X_1_) had a significant effect on the EE %, where by increasing the total Pluronics mixture (X_1_) the EE% decreases as shown by the negative coefficient of X_1_ (Eq. 5). Moreover, the Pluronic P123% (X_2_) had a significant effect on the EE %, where by increasing the Pluronic P123 (X_2_) the EE % increases as shown by its positive coefficient (Eq. 5).

**Table 2:** ANOVA table for the measured responses.

Source	Y1 = EE %					Y2 = PS (nm)				
	Sum of squares	DF	Mean square	F-value	p-value	Sum of squares	DF	Mean square	F-value	p-value
Model	2576.446877	9	286.2718752	221.6502	< 0.0001	196.92	9	21.88	56.05	< 0.0001
X_1_: total polymers	88.22796922	1	88.22796922	68.31181	< 0.0001	3.05	1	3.05	7.81	0.0190
X_2_: P123	7.107040675	1	7.107040675	5.502732	0.0409	109.26	1	109.26	279.88	< 0.0001
X_3_: amount of drug	286.771608	1	286.771608	222.0372	< 0.0001	0.80	1	0.80	2.05	0.1823
X_1_X_2_	10.1498153	1	10.1498153	7.858645	0.0187	2.21	1	2.21	5.66	0.0387
X_1_X_3_	153.8805458	1	153.8805458	119.1443	< 0.0001	0.50	1	0.50	1.29	0.2820
X_2_X_3_	18.90655964	1	18.90655964	14.63868	0.0033	2.77	1	2.77	7.09	0.0238
X_1_^2^	100.5701375	1	100.5701375	77.86792	< 0.0001	1.43	1	1.43	3.67	0.0844
X_2_^2^	1002.940755	1	1002.940755	776.5417	< 0.0001	5.83	1	5.83	14.92	0.0031
X_3_^2^	671.4096312	1	671.4096312	519.8489	< 0.0001	0.44	1	0.44	1.13	0.3131
Residual	12.91547769	10	1.291547769			3.90	10	0.39		
Lack of fit	4.729027686	5	0.945805537	0.577665	0.7192	3.17	5	0.63	4.32	0.0671
Pure error	8.18645	5	1.63729			0.73	5	0.15		
Cor total	2589.362355	19				200.83	19			

The drug amount (X_3_) had a significant effect on the EE %, where by increasing the drug amount (X_3_) the EE % decreased as shown by its negative coefficient (Eq. 5).

As can be deduced from Eq. (5), an interaction effect was present between independent variables. An antagonistic effect was observed between the total Pluronics mixture and Pluronic P123%, whereas a synergistic effect was found between each of the total Pluronics mixture and the drug amount, and the Pluronic P123% and the drug amount, as can be deduced from the positive coefficients of X_13_ and X_23_.

**Figure 1:**
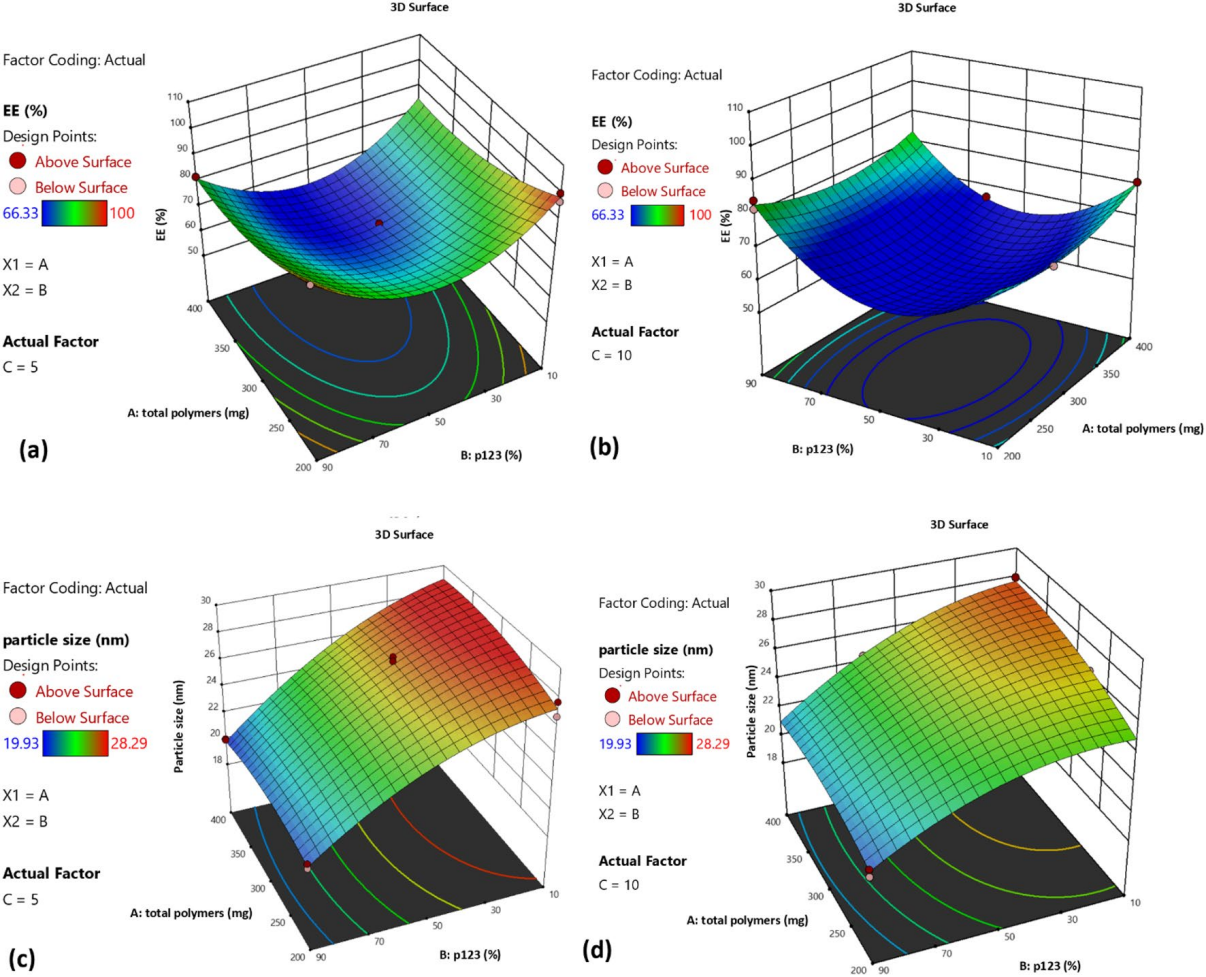
3D response surface plot showing the effect of the total Pluronics mixture (X_1_) and the Pluronic P123% (X_2_) on (**a**) the EE% at low levels of drug amount, (**b**) the EE% at high levels of drug amount, (**c**) the particle size at low levels of drug amount and (**d**) the particle size at high levels of drug amount.

3D surface plots could be used for the understanding of the interaction between factors. As can be observed from Fig. 1a,b, a non-linear relationship was found between the EE% and each of X_1_ and X_2_, at either level of the drug amount. An initial reduction in the EE% was observed with the decrease in the P123%, which was followed by an increase with the further P123% decrease.

### Particle size

As shown in Table 1, the particle size for the prepared formulae ranged from 19.93 to 28.29 nm.

As shown in Table 2, increasing the total Pluronics mixture (X_1_) resulted in a significant increase in the particle size, as shown by its positive coefficient (Eq. 6), whereas increasing the Pluronic P123% (X_2_) resulted in a significant decrease in the particle size, as shown by its negative coefficient (Eq. 6).

As can be deduced from Eq. (6), an interaction effect existed between the independent variables. An antagonistic effect existed between the total Pluronics mixture and Pluronic P123%, as indicated by the negative coefficient of X_12_. Whereas a positive coefficient of X_23_ indicated a synergistic effect between those 2 variables.

The three-D surface plots, as represented in Fig. 1c,d, show a reduction in the particle size with the increase in each of the total Pluronics mixture and the Pluronic P123% as well, at either level of the drug amount.

The PDI for all the prepared formulae were less than 0.25 (results not shown), which indicates the homogeneity of all the prepared formulae.

### Optimization of FA loaded polymeric mixed micelles

Optimization was done using Design Expert® software in order to select the best factors that would result in the formulation of FA loaded micelles with the highest EE % and the smallest particle size. Table 3 shows the composition of the optimized FA micelles formula, along with the predicted and observed responses. The optimized formula was composed of 200 mg total Pluronics mixture, 90% Pluronic P123, and drug amount of 8.58 mg, with a desirability of 0.970. The optimized formula was prepared and characterized in terms of EE% and particle size, in order to calculate the % prediction error. As shown in Table 3, the EE % and the particle size for the optimized formula were 91.00 ± 0.09% and 19.94 ± 0.07 nm, respectively. The prediction error was very low for both responses (Table 3), which indicates the validity of the used design in the preparation and optimization of FA-loaded Pluronics-mixed micelles. Further characterization tests were done on the optimized formula such as the PDI and the zeta potential. The PDI was found to be 0.21 ± 0.05, and the zeta potential was found to be − 3.30 ± 0.05 mv.

**Table 3:** The optimized values of the variables with the predicted and observed responses.

Optimized formula composition	Response	Predicted value	Observed value	Prediction error%*	Desirability
Total pluronics: 200 mg Pluronic P123:90% Drug amount: 8.58 mg	EE (%)	100	91 ± 0.09	9	0.970
	Particle size (nm)	20.02	19.94 ± 0.07	0.59	

*Prediction error = Predicted value – Observed value/Predicted value × 100.

### Characterization of the TPGS-pluronics mixed micelles

The EE%, particle size, PDI, and the zeta potential for the prepared TPGS mixed micelles were 99.89 ± 0.29%, 13.86 ± 0.77 nm, 0.19 ± 0.01 and − 6.02 ± 0.09 mv, respectively. Upon comparing these results with the optimized Pluronic mixed micelles, it is clear that upon incorporation of TPGS, the EE % increased from 91.00 ± 0.09 to 99.89 ± 0.29% (*p-value* < 0.0001), the particle size decreased from 19.94 ± 0.07 nm to 13.86 ± 0.77 nm (*p-value* < 0.0001), and the zeta potential significantly increases from − 3.3 ± 0.05 mv to − 6.02 ± 0.09 mv (*p-value* < 0.0001).

### Differential scanning calorimetry (DSC)

**Figure 2:**
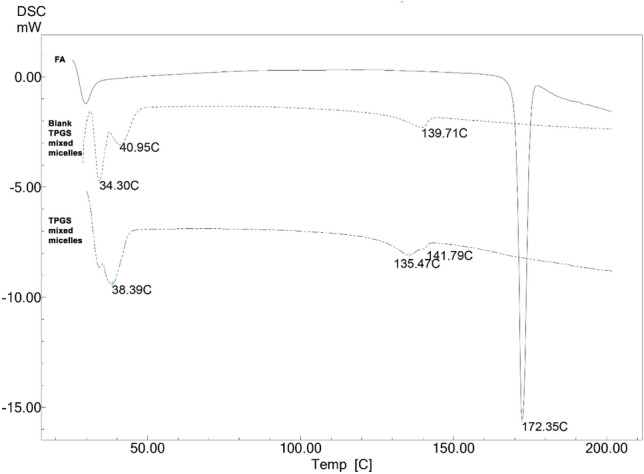
DSC of FA, TPGS mixed micelles, and blank TPGS mixed micelles.

DSC thermogram of FA showed a sharp endothermic peak at 172.35 °C corresponding to its melting point. The thermogram for blank TPGS mixed micelles (Fig. 2) only shows endothermic peaks of polymers. The thermogram of TPGS mixed micelles (O2) shows all endothermic peaks of polymers as shown in the blank TPGS mixed micelles thermogram, but the characteristic peak of FA at 172.35 °C was absent.

### Fourier transform infrared spectroscopy (FTIR)

**Figure 3:**
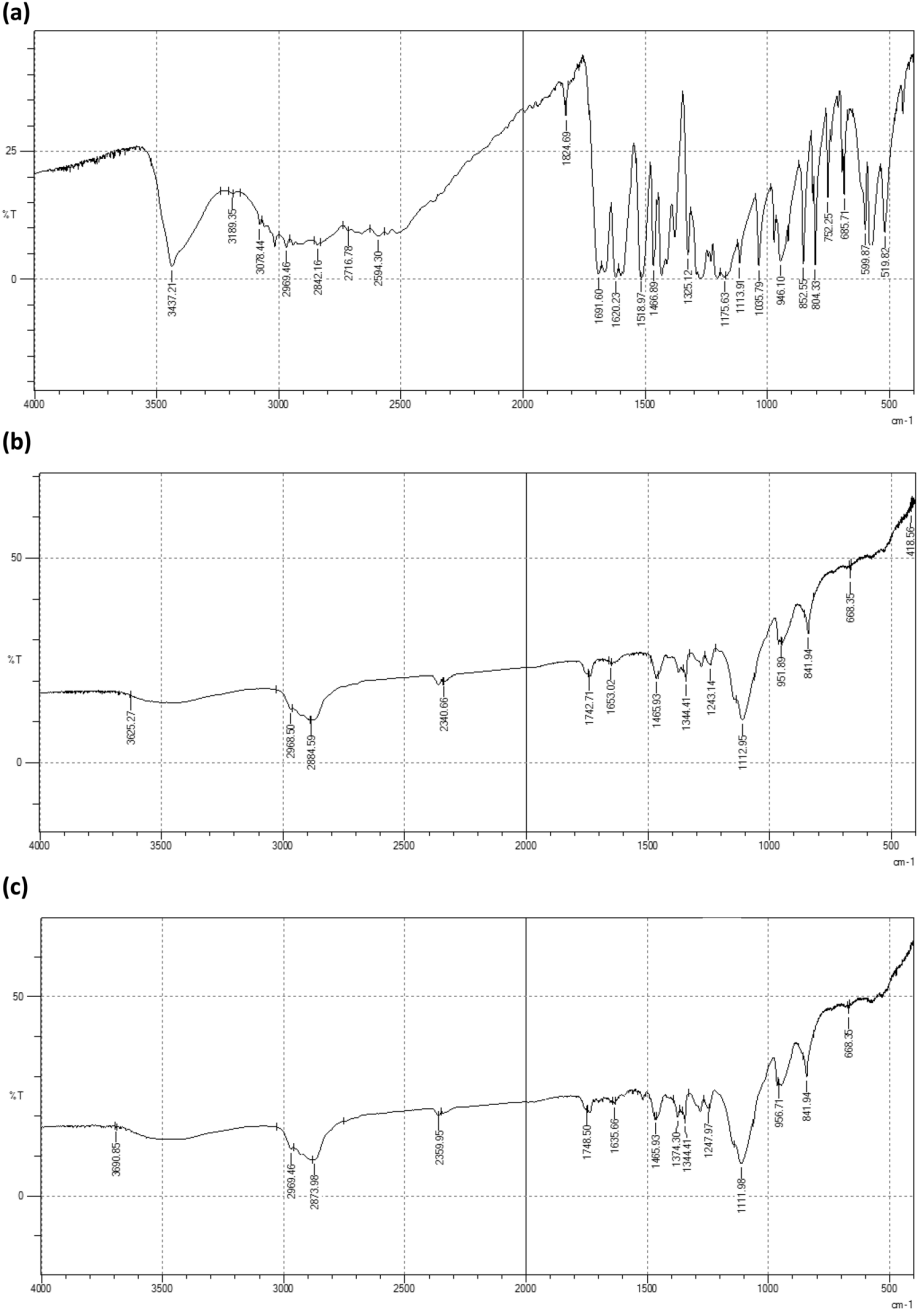
FTIR spectra of (**a**) FA, (**b**) TPGS mixed micelles, (**c**) blank TPGS mixed micelles.

The FTIR spectra of FA, TPGS mixed micelles, and blank TPGS mixed micelles are shown in Fig. 3. The FTIR spectrum of FA revealed characteristics bands at 3437.26 cm^−1^ (corresponding to O–H group)^34^ and 1824 cm^−1^ corresponding to the C=O group^35^. The characteristics peaks of FA were absent in the spectra of TPGS mixed micelles.

### Critical micelle concentration (CMC)

**Figure 4:**
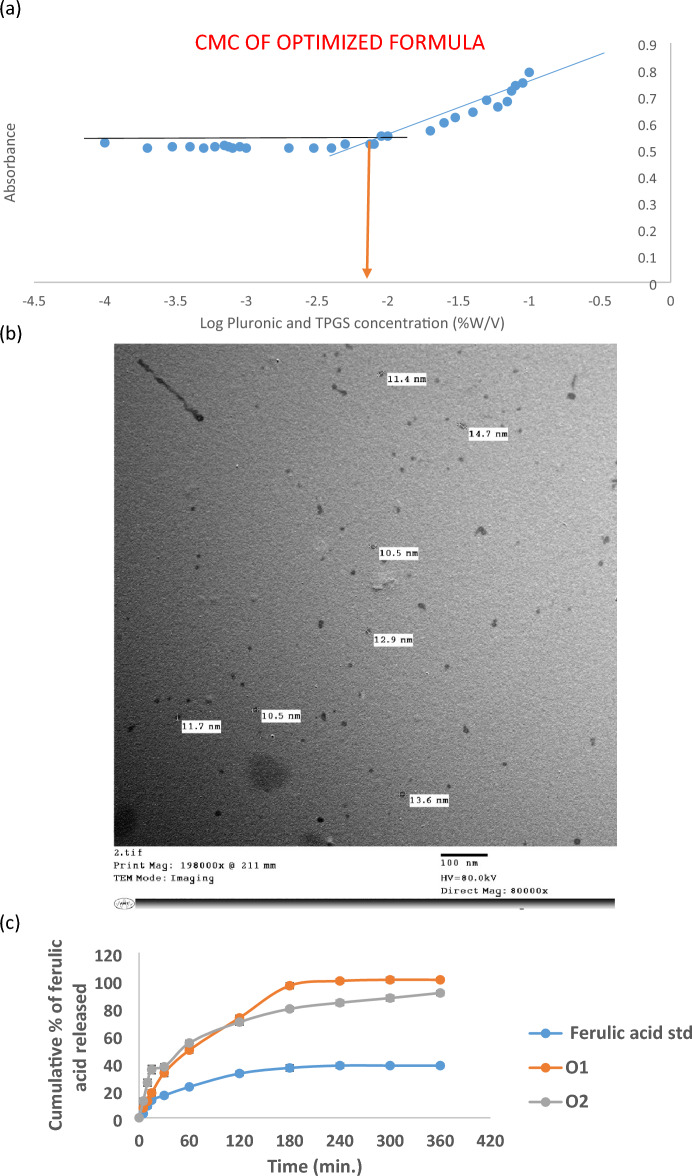
(**a**) Optimized TPGS mixed micelles CMC, (**b**) TEM of FA loaded TPGS mixed micelles, (**c**) Release rate of ferulic acid from O1 and O2 mixed micelles formulae in simulated intestinal condition as compared to the standard FA.

The absorption intensity of iodine was plotted as a function of the polymer concentration as shown in Fig. 4a, in order to determine the CMC of the TPGS-mixed micelles formula. TPGS-mixed micelles formula had a CMC of 0.0075% which is considered a relatively low CMC. Also, the CMC of Pluronic F127, P123, and TPGS were measured and were found to be 0.0025%, 0.0025%, and 0.025%, respectively.

### Transmission electron microscopy (TEM)

The morphology of the TPGS mixed micelles was observed by TEM. As can be seen in Fig. 4b, the TPGS mixed micelles had a smooth surface and a spherical shape. No aggregation was seen as shown in Fig. 4b. The size of the micelles was found to be 10.5 ± 0.12 nm which is slightly smaller than the size measured by the DLS.

### Solubility of ferulic acid in water and polymeric micellar solution

The solubility of FA in water was 40 μg/ml, while in O1 and in O2 was 780 μg/ml and 858 μg/ml, respectively. It can be observed thus, that the solubility of FA was enhanced about 19.5 and 21.5 times, respectively, as compared to that in water.

### In-vitro drug release

Figure 4c shows the release profile of the optimized mixed micelles (O1), the TPGS mixed micelles (O2), and the standard FA, all containing the same drug amount. After 10 min, the % of FA released from the optimized mixed micelles (O1) and the TPGS mixed micelles (O2) were 12.00 ± 2.11% and 25.57 ± 2.52%, respectively, whereas only 8.65 ± 1.55% was released from the standard. After 1 h, 49.15 ± 1.91% and 54.373 ± 1.87% of FA was released from O1 and O2, respectively, whereas only 22.46 ± 1.50% was released from the standard.

After 3 h, the release of FA started to be faster from the optimized formula (O_1_) than from the TPGS mixed micelles (O2), where the release was 96 ± 1.34% versus 79.155 ± 1.22%, respectively, whereas only 36.22 ± 0.99% was released from the standard FA. After 6 h, 100.00 ± 0.25% and 90.72 ± 1.07% of FA was released from the optimized mixed micelles (O_1_) and the TPGS mixed micelles (O2) respectively, whereas only 38.30 ± 0.78% was released from the standard.

### Stability studies

In order to ensure that the prepared TPGS mixed micelles (O2) were of high stability, stability studies were performed according to International Council for Harmonisation of Technical Requirements for Pharmaceuticals for Human Use (ICH), at 4 °C and relative humidity of 55–60% for 3 months^29,36^. Visual examination at monthly intervals revealed no sedimentation, and no particle aggregation. Micelles’particle size, PDI, and EE% measured at monthly intervals showed no significant difference (results not shown). After three months, the results continued to show no significant difference, where the micelles’ particle size was 14.22 ± 0.87, the PDI was 0.201 ± 0.02, and the EE % was 98.03 ± 0.37.

### MTT assay

The cytotoxic effects of B, O2 and FA were evaluated on Vero, Caco-2, PANC-1 MCF-7, HepG-2 and A549 using an MTT assay. Tables S2–S7 showed the cell viability and toxicity after 48 h. The half inhibitory concentration (IC_50_) values for B, O2 and FA were calculated against different cell lines showing a prominent IC_50_ for them against Caco-2 (30.23 µg/ml, 17.1 µg/ml, 191 µg/ml respectively).

### Cell cycle progression and apoptosis

**Figure 5:**
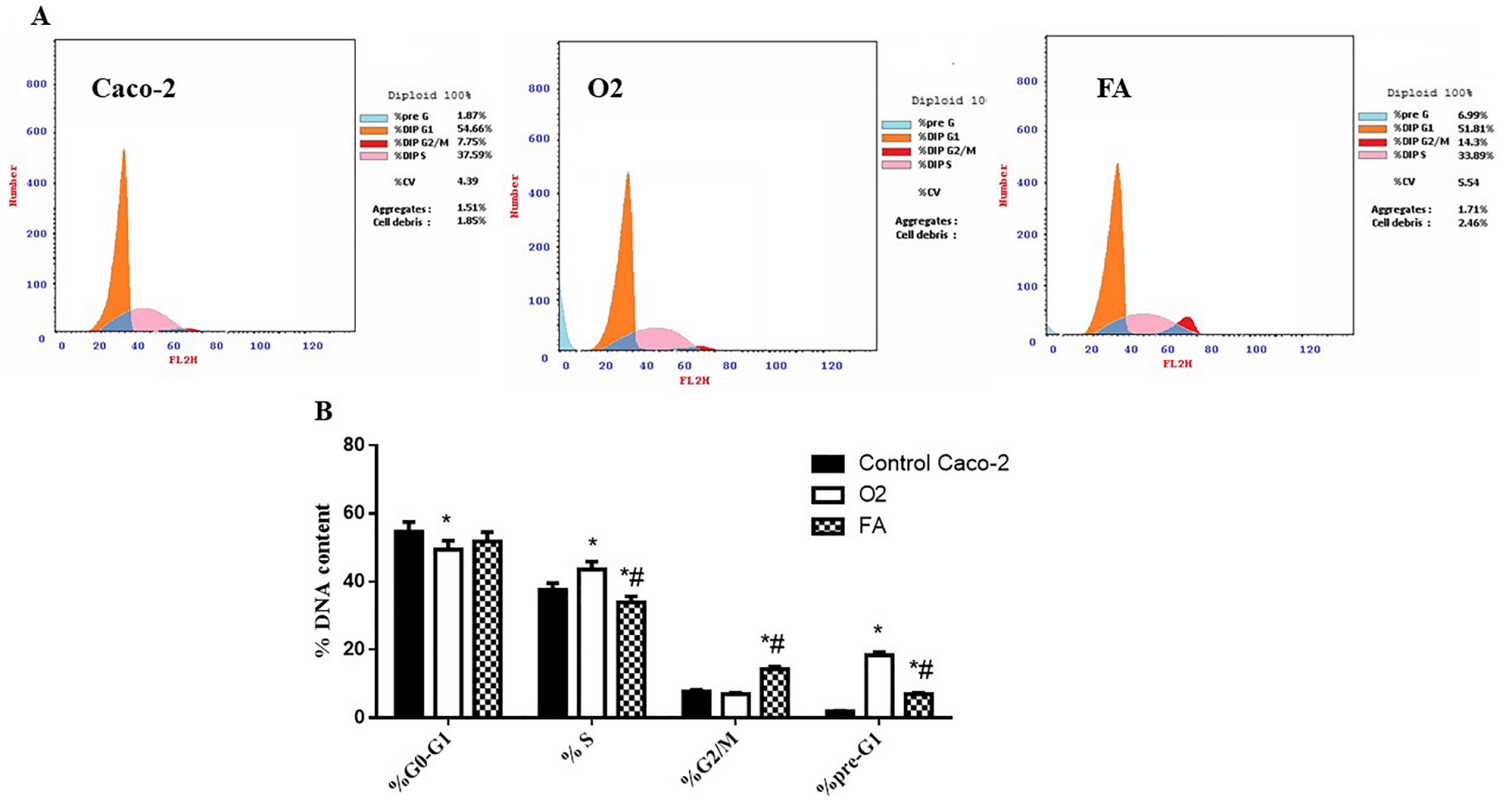
Cell cycle analysis of Caco-2 (untreated) and treated with O2 & FA: (**A**) Histogram showing percentage of cell population in each phase of cell cycle analysis. (**B**) Bar chart showing percentage of cell population in each phase of the cell cycle analysis. *Significance from Caco-2 cells (untreated) at *P* < 0.0001, ^#^Significance from O2 treatment at *P* < 0.0001.

**Figure 6:**
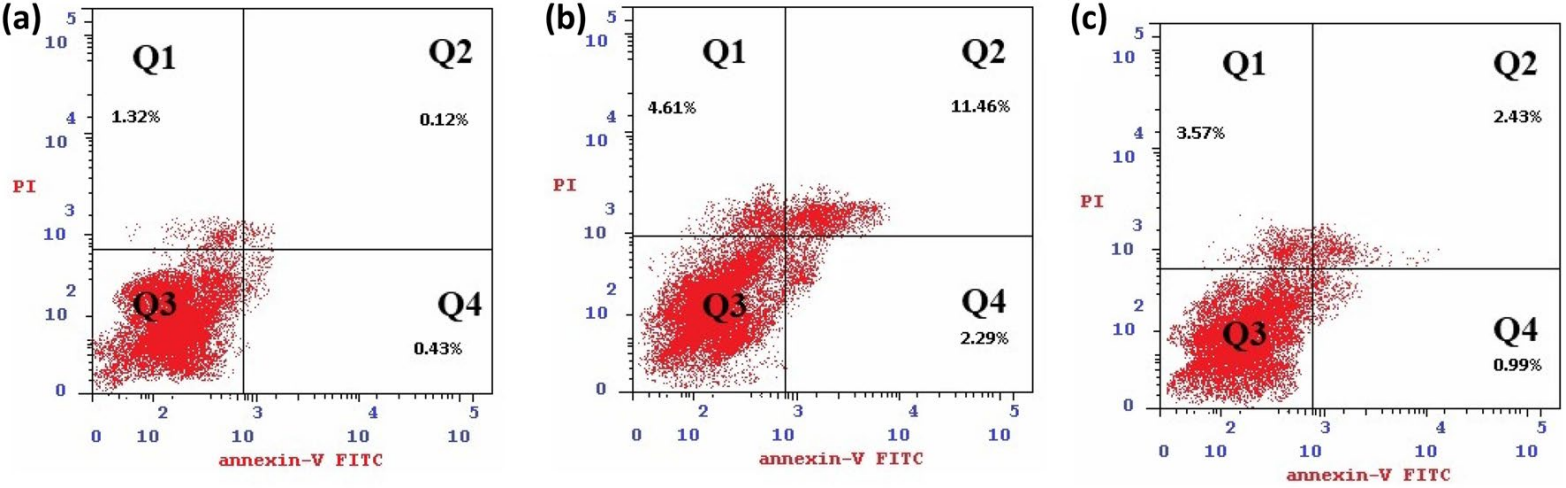
Dot plot representing four quadrant images recorded by flow cytometry analysis for cells stained by Annexin V-FITC and propidium iodide. Q1: shows necrotic cells, Q2: shows later period apoptotic cells, Q3: shows normal cells, Q4: shows early apoptotic cells. (**A**) Control cells, (**B**) O2, (**C**) FA.

Cell cycle analysis revealed that Caco-2 cells treatment with IC50 of TPGS mixed micelles formula (O2) (17.1 µg/ml) resulted in cell cycle arrest at the S phase, whereas treatment with IC50 of FA (191 µg/ml) resulted in cell cycle arrest at G2/M phase and raised apoptosis (Table 4 and Fig. 5). The percentage of Caco-2 cells in the pre-G1 phase has statistically raised from 1.87 to 18.36% after O2 treatment (*P* < 0.0001), and the percentage of Caco-2 cells in G2/M has statistically raised from 7.75% to 14.3% (*P* < 0.0001) after FA treatment. Moreover, after treatment with O2, the Caco-2 cells percentage increased from 37.59 to 43.59% in the S phase, whereas the percentage of Caco-2 cells in S phase has decreased from 37.59% to 33.89% after the treatment with FA. Cell cycle distribution was then detected by flow cytometry. The treatment with O2 showed the highest proportion of cells in the late apoptotic stage (Table 5, Fig. 6).

**Table 4:** Cell cycle analysis of Caco-2 cells following the treatment with IC50 of O2 and FA.

	%G0-G1	%S	%G2/M	%Pre-G1
Control Caco-2	54.66 ± 2.89	37.59 ± 1.98	7.75 ± 0.41	1.87 ± 0.1
O2	49.43 ± 2.61*	43.59 ± 2.31*	6.98 ± 0.37	18.36 ± 0.97*
FA	51.81 ± 2.74	33.89 ± 1.79*^#^	14.3 ± 0.76*^#^	6.99 ± 0.37*^#^

*Significance from Caco-2 cells (untreated) at P < 0.0001.

^#^Significance from O2 treatment at P < 0.0001.

**Table 5:** Concentration of cells (ug/ml) detected at different types of apoptosis induced in Caco-2 cells following treatment with O2 and FA using annexin VFITC/PI staining.

	Total	Early	Late	Necrosis
Control Caco-2	1.87 ± 0.31	0.43 ± 0.02	0.12 ± 0.01	1.32 ± 0.06
O2	18.36 ± 0.83	2.29 ± 0.1	11.46 ± 0.52	4.61 ± 0.21
FA	6.99 ± 1.38	0.99 ± 0.04	2.43 ± 0.11	3.57 ± 0.16

### Regulation of miR-221, Bax, CASP-3 and TP53INP1 expression by O2 and FA

**Figure 7:**
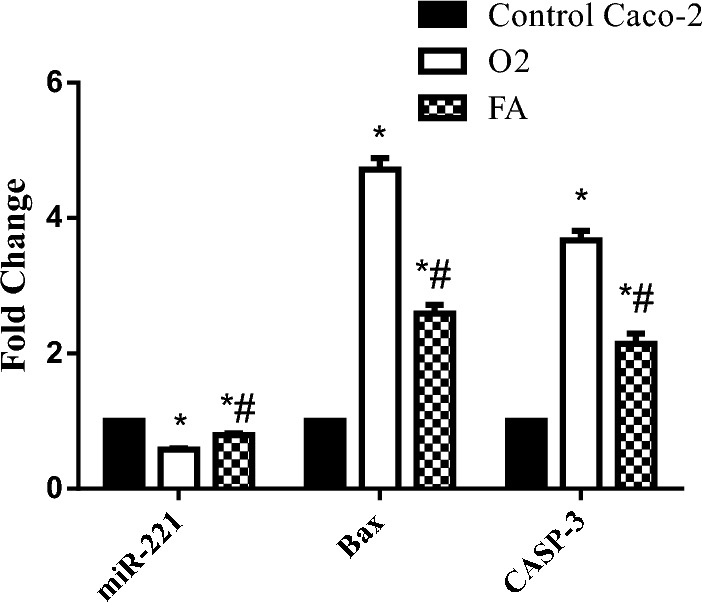
Real-time PCR analysis data depicting the relative normalized expression of miR-221, Bax & CASP-3 after Caco-2 treatment with O2 & FA. *P*-values represented on the graph reflect the statistical significance of various treatments in comparison to untreated cells. The relative expression was calculated based on 2 − ΔΔCt method. *Significance from Caco-2 cells (untreated) at *P* < 0.0001, ^#^Significance from O2 treatment at *P* < 0.05.

**Figure 8:**
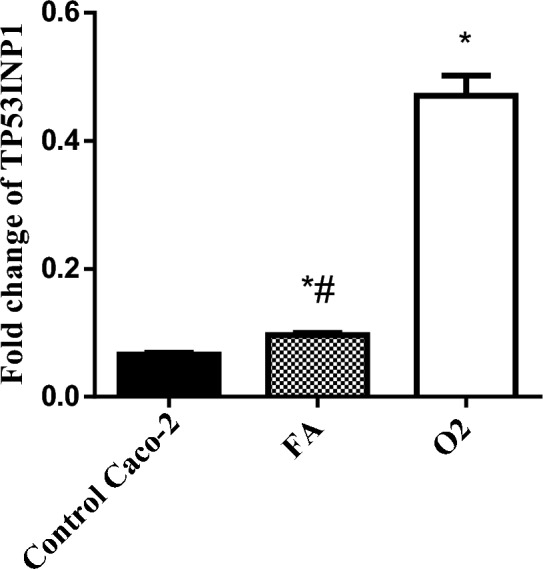
The relative normalized expression of TP53INP1 after treatment of Caco-2 cells with O2, and FA. The relative expression was calculated based on 2 − ΔΔCt method and all data from three separate experiments are shown as mean ± SD. *Significant from Caco-2 control group at P < 0.0001, ^#^Significant from O2 group at P < 0.0001.

RT-PCR results showed that treatment with TPGS mixed micelles formula (O2) down expressed the expression level of miR-221 (0.58 ± 0.02) in Caco-2 cells compared to FA at *P* < 0.05. The expression levels of TP53INP1, Bax and CASP-3 were significantly increased in treated groups with TPGS as compared to the untreated cells at *P* < 0.0001, with a prominent effect for O2 as compared to FA (Table 6, Figs. 7, 8).

**Table 6:** Effect of O2 and FA on miR-221, Bax, CASP-3 expression in Caco-2 cell line.

	miR-221	Bax	CASP-3
Control A549	1	1	1
O2	0.58 ± 0.02*	4.72 ± 0.17*	3.67 ± 0.14*
FA	0.79 ± 0.03*^#^	2.59 ± 0.13*^#^	2.14 ± 0.15*^#^

*Significant from Caco-2 cells (untreated) at *P* < 0.0001.

^#^Significant from O2 treatment at *P* < 0.05.

## Discussion

Many factors were considered in the selection of the suitable components for the formulation of FA-loaded micelles, such as drug encapsulation efficiency, biocompatibility, and the ability to deliver the drug to the cancer cells. It has been reported that using individual Pluronic might not be effective in achieving both high micellar dispersion stability and high encapsulation efficiency of poorly soluble drugs^37^. Combining two different types of Pluronics can therefore overcome these drawbacks.

Pluronic F127 and Pluronic P123 is the most popular combination for the preparation of polymeric mixed micelles, as they can be co-micellized due to their similar numbers of the PPO units^37^.

Pluronic P123, composed of PEO20–PPO68–PEO20, is one of the most commonly used Pluronics. Due to its high proportion of PPO (hydrophobic part), Pluronic P123 is frequently used for the solubilization of hydrophobic drugs. It was also reported to have a significant cytotoxic effect in multidrug-resistant cell lines, as it inhibits the P-glycoprotein drug efflux transport system, which is usually overexpressed in multi-drug resistant cells^38^. However, the low proportion of the PEO (hydrophilic part) leads to the formation of a relatively thin shell of the micelles leading to its poor stability^39^. On the other side, Pluronic F127 (PEO100-PPO69-PEO100) has relatively extended PEO blocks, which is usually used in combination with the Pluronic P123, to increase its stability^36,40^.

A previous study has reported that the combination of two types of Pluronics resulted in enhancing both the drug solubilisation and the micelles’ stability^39^.

The D-optimal design was chosen because it has been reported to reduce the variance associated with the model coefficient estimates^41^.

The high correlation coefficient R^2^ and the reasonable agreement between the adjusted R^2^ and the predicted R^2^, together with the adequate precision values indicate that the models for the two responses can be used to navigate the design space. The high EE% observed with all the formulae might be due to the hydrogen bonding between the ether oxygen of PEO part and the hydroxyl group present in FA^42^.

The decrease in the EE% upon increasing the total Pluronics mixture (X_1_), could be attributed to the increase in the increase in the viscosity of the medium upon increasing the pluronic concentration, resulting in more steric hinderance to the drug to be easily encapsulated^43^.

The increase in the EE % upon increasing the Pluronic P123% (X_2_) could be due to the hydrophobic nature of FA, which has a higher affinity to the Pluronic P123, due its more hydrophobic core (HLB = 7–9), as compared to Pluronic F127 (HLB = 22). This means that the EE % will increase as the Pluronic P123% increases^12,44^.

The decrease in the EE% upon increasing the drug amount (X_3_) could possibly be due to the high drug amount which could have cause a saturation of the micellar core^45^, and thus limit its encapsulation ability^46^. A recent study also reported that upon increasing the drug amount, the EE% decreased^47^.

The 3D plots showed a non-linear relationship between the EE% and each of the total Pluronics mixture (X_1_) and the Pluronic P123% (X_2_), where the EE% showed an initial reduction with the increase in the total Pluronics, which could be attributed to the increase in the viscosity of the medium at the beginning as mentioned earlier. However, the further increase in the Pluronics resulted in an improvement in the EE%, which could be attributed to the formation of more micelles at the high Pluronics concentrations, with the consequence of increasing in the encapsulation of the drug^48^. Moreover, an initial reduction in the entrapment efficiency was observed with increasing the Pluronic P123%, because at low P123% level, the Pluronic F127%, is high, which has a larger molecular weight due to its large hydrophilic heads which is responsible for the steric stabilization of the micelles and more steric hinderance for entrapment of the drug. However, at high levels of Pluronic P123%, the steric hindrance effect of Pluronic F127 would be diminished, leading to an increase in the EE%^12^.

It has been reported that the micelles’ particle size is crucial for achieving a longer circulation time. The particle size must be greater than 10 nm in order to escape kidney filtration, and smaller than 100 nm in order to escape recognition by the Reticulo-endothelial system (RES). This gives the conclusion that the ideal micelles’ particle size should be between 10 and 100 nm^12^.

The particle size for all the prepared formulae reflects the potential of the prepared micelles to escape both the kidney filtration and the RES detection. This, in turn, enhances their accumulation inside the solid tumour via the enhanced permeability and retention effect^11,23,49,50^.

The increase in the particle size upon increasing the total Pluronics mixture (X_1_) could be attributed to the higher viscosity of the micellar dispersion caused by the increased total Pluronics mixture, resulting in an increase in the particle size of the prepared micelles^51^.

The decrease in the particle size upon increasing the Pluronic P123% (X_2_) could be due to the fact that an increase in P123% was accompanied by a reduction in F127, which has a larger molecular weight due to its large hydrophilic heads and is responsible for the steric stabilization of the micelles^12,52^.

The reduction in the particle size due to the increase in the total Pluronics mixture (X_1_) together with the increase in the Pluronic P123% (X_2_) may be due to the increase in the total Pluronics which could lead to a higher micellar layer due to the larger surface active agent layer leading to a reduction in the micellar aggregation, and thus and a decrease in the particle size^53^. This effect was pronounced when Pluronic P123% was increased, as increasing Pluronic P123% resulted in more formation of polymeric mixed micelles encapsulating the drug due to the increase in the hydrophobic polymer, with the consequence of replacing the water molecules, which seem to be existing inside the micelles when there are no drug molecules present in the mixtures^54^.

TPGS (a water-soluble derivative of vitamin E) has gained a wide attention in the recent years due to its many useful applications. It can be useful for solubilizing both water soluble and water insoluble drugs, and most importantly it has been approved as a safe pharmaceutical excipient by the FDA^55^. It is commonly used as a cosurfactant with Pluronics, as it has a synergistic effect with both Pluronic F127 and Pluronic P123^56–60^. In addition, it has been reported to have a potential anticancer effect, which could potentiate the anticancer effect of FA, without causing damage to normal cells^61^.

Accordingly, the optimized micelles formula was selected to be further modified using TPGS. A 1:1 ratio of total Pluronics mixture to TPGS was selected based on preliminary screening (results not shown).

The increase in the EE% upon the incorporation of TPGS could be attributed to the ability of the TPGS to increase the solubility of the poorly soluble drugs^28^ such as FA due to the strong hydrophobic interactions between the TPGS and FA in the micellar core^62^. The decrease in particle size upon the addition of TPGS was due to the hydrophobic interaction between the PPO chains of both P123 and F127 and the hydrophobic parts of TPGS. In addition, TPGS is a surfactant which decreases the surface tension, resulting in a more compact conformation of micelles, with a smaller particle size^26^.

It is well known that a high absolute zeta potential (above 30 mV) value indicates the stability of the micellar dispersion and the less ability to coalescence^63^. However, it has been reported lately that lower zeta potential values were accompanied by an increased potential for cellular uptake^30^.

The low values for zeta potential for both O1 and O2 were expected due to the use of non-ionic Pluronics and TPGS. Also the low values of zeta potential could be attributed to the brush conformation caused by the extension of the hydrophilic PEO segments in the aqueous phase, which in turn causes an outward shift of the slipping plane, at which the zeta potential is measured^44^. These results were in accordance to^23,64^.

The increase in the zeta potential accompanied by the incorporation of TPGS could be attributed to the ability of TPGS to increase in the solubility of FA, yielding more anionic FA in solution^28^. This in turn suggested the higher stability for O2 over O1. A study by Zhao et al., also indicated that increasing TPGS concentration resulted in an increase in the zeta potential of curcumin loaded mixed micelles^42^.

DSC analysis was used to evaluate the physical stability and phase transition behaviour of FA, TPGS mixed micelles, and blank TPGS mixed micelles. Any abrupt or drastic change in the thermal behaviour of either the drug or polymer may indicate possible drug-polymer interaction^65^.

The absence of the endothermic peak of FA in the DSC thermogram of TPGS mixed micelles (O2) confirms that FA was encapsulated inside the micellar core and that it has been converted from the crystalline state to the amorphous state^25^.

The DSC results thus indicated the suitability of these polymers to be used in the prepared formulations. Similar results have been reported^63^.

FTIR was used along with the DSC to investigate the possible physicochemical interactions between the used polymers and FA. The absence of ferulic acid’s distinctive peaks in the spectra of the FA-Loaded TPGS mixed micelles (O2) indicated that the drug was entrapped within the hydrophobic micellar core due to its hydrophobic nature^25,6670^. Thus, the FTIR results together with the DSC confirmed the encapsulation of FA within the micellar core.

The CMC is considered a very important parameter for the determination of the in-vitro and in-vivo stability of the prepared micelles.

The relatively low CMC of TPGS-mixed micelles illustrates the high stability of the prepared micelles and their ability to remain stable after being extensively diluted by the body fluids. Also the CMC of Pluronic F127, P123, and TPGS were in accordance to the values reported in literature^67,68^. The CMC of the TPGS mixed micelles had an intermediate value between the CMC of Pluronic P123 and TPGS. A possible explanation to the low CMC value of the TPGS mixed micelles could be due to attributed to the addition of TPGS, which provided more hydrophobic moiety to the micelles, thus allowing micellization to occur at a lower CMC value. In addition, TPGS might have increased the hydrophobic interactions between the polymer chains in the core of the micelles, which in turn enhances the particles stability^30^. TEM revealed that the TPGS mixed micelles had a smooth surface and a spherical shape which was reported to have better cellular uptake than other shapes such as the rod-shaped micelles. This is due to the fact that the particle curvature of the spherical shaped micelles enhances the contact area with the cell membrane receptors^23^. The size reported by TEM was slightly smaller than the size measured by the DLS. The larger mean size observed by the DLS could be due to the fact that DLS measures the size of particles in solution, meanwhile the TEM measures the size of particles in a dried state^69^.

The in-vitro release of FA from O1 and O2 was studied adopting the dialysis method. In order to achieve sink condition, the dissolution medium was prepared using phosphate buffer saline (pH 7.4) + 0.5% Tween 80^28^.

The initial burst release could be due to the small size of the prepared micelles which is in the nanometre range, which allows the exposure of a large surface area to the dissolution media^70^. Another cause for the initial burst release is the presence of the drug at the interface between the hydrophobic core and the hydrophilic corona of the micelle. This drug may be released by hydration of the interfacial drug molecules and their passive diffusion. Moreover, the presence of hydrophilic polymers in both O1 and O2, speed up the water uptake causing micelles erosion and consequently diffusion of the drug. All these resulted in an increase in the release process which could justify the initial burst release in O1 and O2 as compared to the standard FA^71^.

The faster release profile of TPGS mixed micelles (O2) as compared to the optimized mixed micelles (O1) during the first hour, could be due to the fact that TPGS is a hydrophilic surfactant which helps water to enter into the micelles core, forming more hydrophilic channels, thus allowing faster release of the entrapped drug^28^. The slower release observed in TPGS micelles after 3 h, may be due to the use of TPGS which might have caused an increase in the hydrophobic interactions between the polymer chains in the micelles’ core, resulting in stronger interactions between the drug and the micellar system, and the thus slower the release of FA^30^. Stability studies showed no significant differences after storage for three months at 4 ºC, which validated the stability of the formulation.

To our knowledge this is the first report to evaluate the beneficial effect of TPGS mixed micelles formula in Caco-2 human colorectal cancer cell line. This impact is likely mediated via the down expression of miRNA-221, over expression of Bax, CASP-3 and TP53INP1, inhibition of proliferation and increased apoptosis.

We evaluated the cell viability using MTT assay, flow cytometry, and the expression of Bax, CASP-3, TP53INP1 and miRNA-221 by RT-qPCR, in order to elucidate the mechanism of TPGS mixed micelles formula and ferulic acid in Caco-2 human colorectal cancer cell line.

Our results demonstrated that both ferulic acid and TPGS mixed micelles formula inhibit the growth of the different cancerous cells and decrease cell viability (Tables S2–S7) with the TPGS mixed micelles formula being more effective in cell proliferation inhibition especially on Caco-2 human colorectal cancer cell line (Table S3). A possible cause for the significantly enhanced cytotoxic effect of the optimized formula is the presence of a high amount of Pluronic P123 which could have a synergistic cytotoxic effect with ferulic acid, as it has been reported to increase cytotoxicity and inhibit P-glycoprotein (P-gp). Pluronic copolymers were reported to enhance the antimetastatic effects when they have HLB ranging from 8 to 16.

The downregulation of matrix metalloproteinase-9 (MMP-9) was a contributing factor in the metastatic action of P123 in both in-vitro and in-vivo. Therefore, Pluronic P123 with an HLB of 8 could be a potential excipient for drug delivery systems in order to inhibit cancer metastasis^72^.

A second cause for the significantly enhanced cytotoxic effect of the optimized formula was the presence of TPGS, which is also one of the P-gp inhibitory excipients. A previous study found out that the incorporation of TPGS with Pluronic P123 resulted in an enhancement in the in-vitro cytotoxicity effect. Additionally, it was claimed that the anticancer effect of TPGS was linked to its special apoptosis-inducing capabilities via the generation of reactive oxygen species (ROS). ROS have the potential to harm cells' DNA, proteins, and fatty acids, which would cause apoptotic cell death^56^.

In agreement with our results, a study performed by^73^ found that IC50 of Ferulic acid at 154 µg/ml which cause half cells of HCT-15 were dead. Moreover the same effect was observed by a study carried out by^6^ who found that the ferulic acid inhibited cell proliferation of breast cancer cell line MDA-MB-231 in a dose dependent aspect.

Our results suggested that FA inhibited the proliferation of Caco-2 cell line by cell cycle arrest at G2/M phase and raised the percentage of Caco-2 cells at S phase. On the other hand, TPGS mixed micelles formula induced cell cycle arrest at S phase (Tables 4, 5 and Figs. 5, 6). This comes in agreement with a study carried by^74^ who found that FA treatment of HCT-116 cells with different concentrations for 48 h decreased the S phase compared to vehicle-treated cells.

We investigated the inhibitory effect of FA and TPGS mixed micelles formula on carcinogenesis which is mediated through several signalling pathways as miRNA-221, Bax, CASP-3 and TP53INP1. Our results revealed that IC50 of FA and TPGS mixed micelles formula significantly increased the expression level of CASP-3, with a more prominent effect of TPGS mixed micelles formula as compared to FA treatment. The same effect was observed by a study carried out by^6^ where CASP-3 activity was enhanced by FA treatment in breast cancer cell line MDA-MB-231.

A group of small non-coding RNA molecules called microRNAs (miRNAs) are found in eukaryotes and are involved in the post-transcriptional, translational, and RNA silencing processes that control the expression of genes. miRNAs have been linked to the development and spread of numerous malignancies, including brain, lung, breast, liver, prostate, and colorectal cancer.^75^.

The expression level of miRNA-221 was significantly down expressed upon treatment of the Caco-2 cell line by IC50 of TPGS mixed micelles formula and FA. In accordance with our study^76^ found that miR-221-5p was significantly downregulated in the atrial fibrillation group. The gene expression level of TP53INP1, was overexpressed after treatment of the Caco-2 cell line by TPGS mixed micelles formula. This might be attributed to the fact that miR‑221inhibited the autophagy activity, which, in turn, promoted the cell survival in colorectal cancer. Autophagy is a process in which cells are degraded by the elimination of the damaged or superfluous proteins, and unnecessary or dysfunctional cellular components. Meanwhile TP53INP1 is considered one of the regulators of autophagy in which TP53INP1 protein was a target of miR‑221 in CRC cells, which might elucidate the inhibitory effect of autophagy by miR‑221^1^.

## Conclusion

In the present study, FA mixed polymeric micelles were prepared using the thin film hydration method, and optimized using D-optimal design, to have the maximum entrapment efficiency with a small and uniform particle size. Statistical analysis of the D-optimal design showed significant models for both the EE% and the PS. Model analysis showed that the total Pluronic mixture (X_1_) and Pluronic P123% (X_2_) had significant effects on EE% and PS, while drug amount (X_3_) had a significant effect only on the EE%. The optimized FA mixed polymeric micelles formula, with a desirability of 0.970, was chosen and evaluated. The TPGS mixed micelles was further prepared and showed an EE% of 99.89 ± 0.29%, a particle size of 13.86 ± 0.77 nm, a PDI of 0.19 ± 0.01, and a zeta potential of − 6.02 ± 0.09 mv. The saturation solubility of TPGS mixed micelles was approximately 21.5 times that of free FA in water. Transmission electron microscopy revealed spherical shaped globules with smooth surface. In-vitro drug release studies showed notable increase in the release rate of FA from the optimized formula, as compared to the corresponding standard FA. The cytotoxic effects of TPGS mixed micelles formula and FA were evaluated against different cell lines. IC_50_ values were calculated showing a prominent IC50 for them against Caco-2. TPGS mixed micelles formula down expressed the expression level of miR-221 and increased the expression of Bax and CASP-3 in Caco-2 cells compared to FA. Moreover, the gene level of TP53INP1 in the cells exposed to FA from TPGS mixed micelles formula was significantly increased compared to free FA.

The optimized formula loaded with FA showed a significant cytotoxicity improvement, cell proliferation and apoptosis, and autophagy induction in the colorectal cancer Caco-2 cell line. Thus, FA-loaded TPGS mixed micelles could be a potential therapeutic agent for colorectal cancer by targeting miRNA-221/TP53INP1 axis-mediated autophagy.

### Supplementary Information

Supplementary Tables.

## Data Availability

The datasets used and/or analysed during the current study available from the corresponding author on reasonable request.
