# Supplementary material for: An approach for an enhanced anticancer activity of ferulic acid-loaded polymeric micelles via MicroRNA-221 mediated activation of TP53INP1 in caco-2 cell line
Source: Sci Rep. 2024 Jan 24;14:2073. doi: 10.1038/s41598-024-52143-y (PMC10808409; doi:10.1038/s41598-024-52143-y)
Supplement: Supplementary file 1 — Supplementary Tables. [file 41598_2024_52143_MOESM1_ESM.docx]

**S 1: The sequence of primer used in the study**

| Target gene | Primer name | Primer sequence from (5' to 3') |
| --- | --- | --- |
| Bax | Bax F  Bax R | 5’-GTTTCA TCC AGG ATC GAG CAG-3’  5’-CATCTT CTT CCA GAT GGT GA-3’ |
| CASP-3 | CASP-3 F  CASP-3 R | 5’-TTC ATT ATT CAG GCC TGC CGA GG-3’  5’-TTC TGA CAG GCC ATG TCA TCC TCA-3’ |
| β-actin | β-actin F  β-actin R | 5’-GTGACATCCACACCCAGAGG-3’  5’-ACAGGATGTCAAAACTGCCC-3’ |

**S2: Cytotoxicity evaluation of B, O2 and FA at various concentrations against Vero cell line**

| ID | Conc. (ug/ml) | Mean O.D | Viability % | Toxicity % | IC50 (µg/ml) |
| --- | --- | --- | --- | --- | --- |
| Vero | --- | 0.385 ± 0.006 | 100 | 0 |  |
| B | 500 | 0.02 ± 0.0005 | 5.19 | 94.8 | 88.19 |
|  | 250 | 0.021 ± 0.001 | 5.45 | 94.5 |  |
|  | 125 | 0.083 ± 0.009 | 21.5 | 78.4 |  |
|  | 62.5 | 0.26 ± 0.004 | 68.6 | 31.3 |  |
|  | 31.25 | 0.38 ± 0.005 | 99.6 | 0.34 |  |
|  | 15.62 | 0.38 ± 0.008 | 99.7 | 0.25 |  |
| O2 | 500 | 0.19 ± 0.0003 | 5.1 | 94.9 | 43.5 |
|  | 250 | 0.02 ± 0.001 | 5.36 | 94.6 |  |
|  | 125 | 0.022 ± 0.001 | 5.88 | 94.1 |  |
|  | 62.5 | 0.09 ± 0.008 | 23.4 | 76.5 |  |
|  | 31.25 | 0.231 ± 0.008 | 60 | 40 |  |
|  | 15.62 | 0.38 ± 0.008 | 99.6 | 0.34 |  |
| FA | 1000 | 0.033 ± 0.004 | 8.74 | 91.25 | 360 |
|  | 500 | 0.11 ± 0.002 | 28.5 | 71.5 |  |
|  | 250 | 0.24 ± 0.01 | 63.3 | 36.6 |  |
|  | 125 | 0.35 ± 0.004 | 91.86 | 8.13 |  |
|  | 62.5 | 0.38 ± 0.004 | 100 | 0 |  |
|  | 31.25 | 0.38 ± 0.002 | 100 | 0 |  |

**S3: Cytotoxicity evaluation of B, O2 and FA at various concentrations against Caco-2 cell line**

| ID | Conc. (ug/ml) | Mean O.D | Viability % | Toxicity % | IC50 (µg/ml) |
| --- | --- | --- | --- | --- | --- |
| Caco-2 | --- | 0.357 ± 0.008 | 100 | 0 |  |
| B | 500 | 0.019 ± 0.0003 | 5.41 | 94.5 | 30.23 |
|  | 250 | 0.02 ± 0.001 | 5.6 | 94.4 |  |
|  | 125 | 0.021 ± 0.001 | 5.97 | 94 |  |
|  | 62.5 | 0.03 ± 0.002 | 10.5 | 89.4 |  |
|  | 31.25 | 0.109 ± 0.009 | 30.6 | 69.4 |  |
|  | 15.62 | 0.29 ± 0.003 | 82.5 | 17.5 |  |
| O2 | 500 | 0.018 ± 0.0003 | 5.1 | 94.9 | 17.1 |
|  | 250 | 0.018 ± 0.0003 | 5.22 | 94.7 |  |
|  | 125 | 0.021 ± 0.001 | 5.88 | 94.1 |  |
|  | 62.5 | 0.02 ± 0.002 | 6.25 | 93.7 |  |
|  | 31.25 | 0.079 ± 0.007 | 22.3 | 77.6 |  |
|  | 15.62 | 0.21 ± 0.004 | 60.1 | 39.9 |  |
| FA | 1000 | 0.018 ± 0.001 | 5.04 | 94.95 | 191 |
|  | 500 | 0.038 ± 0.007 | 10.6 | 89.3 |  |
|  | 250 | 0.11 ± 0.006 | 31.9 | 68.1 |  |
|  | 125 | 0.246 ± 0.008 | 68.9 | 31.1 |  |
|  | 62.5 | 0.33 ± 0.005 | 93.9 | 6.1 |  |
|  | 31.25 | 0.355 ± 0.004 | 99.4 | 0.56 |  |

**S4: Cytotoxicity evaluation of B, O2 and FA at various concentrations against PANC-1 cell line**

| ID | Conc. (ug/ml) | Mean O.D | Viability % | Toxicity % | IC50 (µg/ml) |
| --- | --- | --- | --- | --- | --- |
| PANC-1 | --- | 0.296 ± 0.006 | 100 | 0 |  |
| B | 500 | 0.018 ± 0.0003 | 6.30 | 93.7 | 45.3 |
|  | 250 | 0.019 ± 0.0003 | 6.5 | 93.4 |  |
|  | 125 | 0.03 ± 0.002 | 12.38 | 87.6 |  |
|  | 62.5 | 0.09 ± 0.006 | 31.6 | 68.3 |  |
|  | 31.25 | 0.167 ± 0.008 | 56.6 | 43.3 |  |
|  | 15.62 | 0.285 ± 0.01 | 96.3 | 3.60 |  |
| O2 | 500 | 0.018 ± 0.0003 | 6.19 | 93.8 | 34.8 |
|  | 250 | 0.019 ± 0.0003 | 6.53 | 93.5 |  |
|  | 125 | 0.020 ± 0.0008 | 6.98 | 93.01 |  |
|  | 62.5 | 0.068 ± 0.008 | 23.2 | 76.8 |  |
|  | 31.25 | 0.134 ± 0.004 | 45.3 | 54.7 |  |
|  | 15.62 | 0.226 ± 0.006 | 76.3 | 23.6 |  |
| FA | 1000 | 0.018 ± 0.0003 | 6.30 | 93.7 | 333 |
|  | 500 | 0.067 ± 0.005 | 22.7 | 77.25 |  |
|  | 250 | 0.16 ± 0.004 | 55.85 | 44.1 |  |
|  | 125 | 0.281 ± 0.009 | 95.15 | 4.84 |  |
|  | 62.5 | 0.294 ± 0.004 | 99.5 | 0.45 |  |
|  | 31.25 | 0.297 ± 0.003 | 100 | 0 |  |

**S5: Cytotoxicity evaluation of B, O2 and FA at various concentrations against MCF-7 cell line**

| ID | Conc. (ug/ml) | Mean O.D | Viability % | Toxicity % | IC50 (µg/ml) |
| --- | --- | --- | --- | --- | --- |
| MCF-7 | --- | 0.34 ± 0.006 | 100 | 0 |  |
| B | 500 | 0.018 ± 0.0003 | 5.47 | 94.5 | 49.7 |
|  | 250 | 0.02 ± 0.001 | 5.86 | 94.1 |  |
|  | 125 | 0.019 ± 0.0008 | 5.66 | 94.3 |  |
|  | 62.5 | 0.104 ± 0.006 | 30.5 | 69.5 |  |
|  | 31.25 | 0.26 ± 0.003 | 78.6 | 21.3 |  |
|  | 15.62 | 0.343 ± 0.004 | 100 | 0 |  |
| O2 | 500 | 0.019 ± 0.0008 | 5.66 | 94.3 | 37.3 |
|  | 250 | 0.019 ± 0.0005 | 5.57 | 94.4 |  |
|  | 125 | 0.019 ± 0.0003 | 5.76 | 94.23 |  |
|  | 62.5 | 0.074 ± 0.006 | 21.7 | 78.3 |  |
|  | 31.25 | 0.144 ± 0.003 | 42.2 | 57.8 |  |
|  | 15.62 | 0.305 ± 0.005 | 89.4 | 10.6 |  |
| FA | 1000 | 0.021 ± 0.001 | 6.35 | 93.6 | 202 |
|  | 500 | 0.065 ± 0.006 | 19.1 | 80.9 |  |
|  | 250 | 0.116 ± 0.005 | 34.01 | 65.9 |  |
|  | 125 | 0.257 ± 0.003 | 75.5 | 24.5 |  |
|  | 62.5 | 0.34 ± 0.007 | 99.7 | 0.29 |  |
|  | 31.25 | 0.34 ± 0.004 | 99.7 | 0.3 |  |

**S6: Cytotoxicity evaluation of B, O2 and FA at various concentrations against HepG-2 cell line**

| ID | Conc. (ug/ml) | Mean O.D | Viability % | Toxicity % | IC50 (µg/ml) |
| --- | --- | --- | --- | --- | --- |
| HepG-2 | --- | 0.364 ± 0.005 | 100 | 0 |  |
| B | 500 | 0.02 ± 0.0005 | 5.49 | 94.5 | 42.9 |
|  | 250 | 0.02 ± 0.001 | 6.41 | 93.6 |  |
|  | 125 | 0.02 ± 0.001 | 6.5 | 93.5 |  |
|  | 62.5 | 0.07 ± 0.008 | 19.9 | 80.1 |  |
|  | 31.25 | 0.22 ± 0.006 | 61.8 | 38.2 |  |
|  | 15.62 | 0.366 ± 0.005 | 100 | 0 |  |
| O2 | 500 | 0.02 ± 0.0005 | 5.49 | 94.5 | 36.01 |
|  | 250 | 0.02 ± 0.001 | 5.49 | 94.5 |  |
|  | 125 | 0.02 ± 0.001 | 6.5 | 93.5 |  |
|  | 62.5 | 0.034 ± 0.003 | 9.52 | 90.5 |  |
|  | 31.25 | 0.15 ± 0.003 | 43.04 | 56.9 |  |
|  | 15.62 | 0.34 ± 0.01 | 95.1 | 4.9 |  |
| FA | 1000 | 0.021 ± 0.001 | 5.76 | 94.2 | 116 |
|  | 500 | 0.036 ± 0.002 | 9.89 | 90.1 |  |
|  | 250 | 0.048 ± 0.004 | 13.2 | 86.7 |  |
|  | 125 | 0.146 ± 0.006 | 40.1 | 59.9 |  |
|  | 62.5 | 0.35 ± 0.003 | 97.5 | 2.5 |  |
|  | 31.25 | 0.36 ± 0.009 | 98.9 | 1.1 |  |

**S7: Cytotoxicity evaluation of B, O2 and FA at various concentrations against A549 cell line.**

| ID | Conc. (ug/ml) | Mean O.D | Viability % | Toxicity % | IC50 (µg/ml) |
| --- | --- | --- | --- | --- | --- |
| A549 | --- | 0.275 ± 0.007 | 100 | 0 |  |
| B | 500 | 0.018 ± 0.0003 | 6.66 | 93.3 | 49.4 |
|  | 250 | 0.019 ± 0.0003 | 7.03 | 92.9 |  |
|  | 125 | 0.02 ± 0.001 | 7.75 | 92.2 |  |
|  | 62.5 | 0.08 ± 0.004 | 31.4 | 68.6 |  |
|  | 31.25 | 0.21 ± 0.005 | 74.9 | 25.1 |  |
|  | 15.62 | 0.27 ± 0.005 | 100 | 0 |  |
| O2 | 500 | 0.021 ± 0.001 | 7.64 | 92.4 | 37.5 |
|  | 250 | 0.022 ± 0.001 | 8 | 92 |  |
|  | 125 | 0.032 ± 0.003 | 11.9 | 88.1 |  |
|  | 62.5 | 0.064 ± 0.004 | 23.3 | 76.7 |  |
|  | 31.25 | 0.13 ± 0.006 | 47.6 | 52.4 |  |
|  | 15.62 | 0.22 ± 0.005 | 83.03 | 16.9 |  |
| FA | 1000 | 0.023 ± 0.001 | 8.6 | 91.4 | 308 |
|  | 500 | 0.058 ± 0.005 | 21.2 | 78.8 |  |
|  | 250 | 0.12 ± 0.01 | 44.4 | 55.6 |  |
|  | 125 | 0.24 ± 0.004 | 88.9 | 11.03 |  |
|  | 62.5 | 0.272 ± 0.012 | 98.9 | 1.1 |  |
|  | 31.25 | 0.27 ± 0.008 | 100 | 0 |  |
